# Intestinal parasites, growth and physical fitness of schoolchildren in poor neighbourhoods of Port Elizabeth, South Africa: a cross-sectional survey

**DOI:** 10.1186/s13071-016-1761-5

**Published:** 2016-09-05

**Authors:** Ivan Müller, Peiling Yap, Peter Steinmann, Bruce P. Damons, Christian Schindler, Harald Seelig, Nan S. N. Htun, Nicole Probst-Hensch, Markus Gerber, Rosa du Randt, Uwe Pühse, Cheryl Walter, Jürg Utzinger

**Affiliations:** 1 Swiss Tropical and Public Health Institute, P.O. Box, , CH-4002 Basel, Switzerland; 2 University of Basel, P.O. Box, , CH-4003 Basel, Switzerland; 3 Department of Sport, Exercise and Health, University of Basel, St. Jakobsturm, Birsstrasse 320B, CH-4056 Basel, Switzerland; 4 Institute of Infectious Disease and Epidemiology, Tan Tock Seng Hospital, 308433 Singapore, Singapore; 5 Faculty of Education, Nelson Mandela Metropolitan University, P.O. Box 77000, Port Elizabeth, 6031 South Africa; 6 Department of Human Movement Science, Nelson Mandela Metropolitan University, P.O. Box 77000, Port Elizabeth, 6031 South Africa

**Keywords:** Anthropometric indicators, Haemoglobin, Intestinal polyparasitism, Intestinal protozoa, Physical fitness, Soil-transmitted helminths, South Africa

## Abstract

**Background:**

As traditional lifestyle and diets change with social and economic development, disadvantaged communities in low- and middle-income countries increasingly face a double burden of communicable and non-communicable diseases. We studied the relationship between physical fitness and infections with soil-transmitted helminths (STHs), intestinal protozoa and *Helicobacter pylori* among schoolchildren in Port Elizabeth, South Africa.

**Methods:**

We conducted a cross-sectional survey among 1009 children, aged 9 to 12 years, from eight primary schools in socioeconomically disadvantaged neighbourhoods of Port Elizabeth. Physical fitness was determined using field-deployable tests of the Eurofit fitness test battery. Stool samples were analysed with the Kato-Katz thick smear technique to diagnose STHs and with rapid diagnostic tests (RDTs) to detect intestinal protozoa and *H. pylori* infections. Haemoglobin (Hb) levels were assessed and anthropometric indicators determined.

**Results:**

Complete data were available for 934 children (92 %). In two schools, high STH prevalences were found (*Ascaris lumbricoides* 60 and 72 %; *Trichuris trichiura* 65 % each). For boys and girls co-infected with *A. lumbricoides* and *T. trichiura* (*n* = 155) the maximal oxygen uptake (VO_2_ max) was estimated to be 50.1 and 47.2 ml kg^-1^ min^-1^, compared to 51.5 and 47.4 ml kg^-1^ min^-1^ for their non-infected peers (*n* = 278), respectively. On average, children without helminth infections had greater body mass (*P* = 0.011), height (*P* = 0.009) and a higher body mass index (*P* = 0.024) and were less often stunted (*P* = 0.006), but not significantly less wasted compared to their peers with a single or dual species infection. Among 9-year-old boys, a negative correlation between helminth infections and VO_2_ max, grip strength and standing broad jump distance was observed (*P* = 0.038). The overall mean Hb level was 122.2 g l^-1^. In the two schools with the highest prevalence of STHs the Hb means were 119.7 and 120.5 g l^-1^, respectively.

**Conclusions:**

Intestinal parasite infections appear to have a small but significant negative effect on the physical fitness of infected children, as expressed by their maximal oxygen uptake. We observed a clear impact on anthropometric indicators.

**Electronic supplementary material:**

The online version of this article (doi:10.1186/s13071-016-1761-5) contains supplementary material, which is available to authorized users.

## Background

Globally, more than 1 billion people are infected with soil-transmitted helminths (STHs; *Ascaris lumbricoides*, hookworms and *Trichuris trichiura*) and *Schistosoma* spp. [1–3]. The symptoms most frequently associated with these parasitic worm infections include abdominal pain, diarrhoea, anaemia, growth retardation and cognitive impairment [4], ultimately resulting in reduced physical fitness and work productivity [5]. Important risk factors for STH and *Schistosoma* spp. infections are a lack of clean water, sanitation and hygiene (WASH) [6, 7]. Permissive conditions are commonly found in socioeconomically deprived neighbourhoods in low- and middle-income countries, including in South Africa [8]. Intestinal protozoa such as *Cryptosporidium parvum*, *Entamoeba* spp. and *Giardia intestinalis* are associated with poor living conditions [9]. Their transmission mostly occurs through faecal contamination of food and water [10]. They may lead to symptoms such as abdominal pain, diarrhoea and nausea. Many low- and middle-income countries struggle to control such infectious diseases stemming from traditional challenges [11]. In South Africa, a country that shows considerable health inequity in global terms (e.g. Gini index of 0.63 in 2011 [12]), socioeconomically deprived communities with a high burden of infectious diseases live in close proximity to affluent ones with a disease burden profile typical of western societies. Among both populations, non-communicable diseases (e.g. diabetes, cardiovascular- and obesity-related conditions and cancers) are rapidly increasing, fuelled by unhealthy lifestyles including poor nutritional habits and sedentary lifestyles [13]. South Africa’s 2014 Report Card on Physical Activity for Children and Youth [14] highlights the current concerns for the health and well-being of children and youth in relation to declining physical activity levels and increasing rates of consumption of soft-drinks and fast food.

Low levels of in-school physical activity have been documented for children in Port Elizabeth in the frame of a study by Walter et al. [15] who focused on primary schoolchildren in disadvantaged schools. Low quality and often inaccessible sport and recreation facilities, a lack of qualified teachers and an irregular physical education schedule complicate the promotion of age-appropriate physical activity among schoolchildren at disadvantaged schools. The resulting dual burden of diseases (i.e. non-communicable chronic conditions and infectious diseases) puts children at an increased risk of compromised health that may hamper their development, wellbeing and future prospects [13, 16, 17]. Moreover, this dual burden is a challenge for the health system.

The “Disease, Activity and Schoolchildren’s Health” (DASH) study in Port Elizabeth, South Africa, aims to investigate this dual disease burden (i.e. non-communicable chronic conditions and infectious diseases) among children in selected primary schools located in disadvantaged neighbourhoods [18]. Here, we report the findings pertaining to parasite infections and physical activity from a cross-sectional survey among 9- to 12-year-old children. The objectives of this cross-sectional survey were (i) to determine the prevalences of intestinal parasite infections and *Helicobacter pylori*; (ii) to assess the haemoglobin (Hb) levels and anthropometric indicators; (iii) to comprehensively measure the physical fitness levels; and (iv) to investigate possible associations between infection status and other measured variables.

## Methods

### Study site and school selection

The study was carried out at eight primary schools in socioeconomically disadvantaged neighbourhoods of Port Elizabeth, in the Western region of the Eastern Cape province of South Africa (geographical coordinates: 34°07′54″S to 33°57′29″S latitude and 25°36′00″E to 25°55′49″E longitude, altitude: extends from 0 m to approximately 100 m above sea level) in February 2015. The study population consisted of coloured children (of mixed race ancestry, and generally Afrikaans speaking) and black African children (largely Xhosa speaking), residing in areas previously demarcated for these specific race groups, in accordance with past Apartheid legislation. Colloquially, these respective areas are referred to as the northern areas (for coloured people) and townships (for black African people). The people living in these areas are still detrimentally affected by the legacy of Apartheid [19, 20]. A total of 103 quintile three primary schools (where quintile one denotes the poorest and quintile five the “least poor” schools, with the degree of poverty referring to the neighbourhood around school locations) were contacted to explore interest in study participation. Positive responses were received from 25 schools. Eight schools were finally included in the study, with selection based on (i) size in terms of the number of students; (ii) geographical location; (iii) representation of the different target communities; and (iv) commitment to support the project activities.

### Study design

The DASH study is a cohort study with a physical intervention component to determine whether WASH and an education and nutrition programme can reduce the prevalence of parasitic infections and improve physical fitness levels among 9- to 12-year-old children [18]. A single stool and a single urine sample were collected for parasitological work-up to diagnose helminth and intestinal protozoa infections using light microscopy. Anthropometric indicators (i.e. height and weight) and Hb concentrations were assessed by trained examiners or nurses. Physical fitness was determined by measuring the participants’ performance in a grip strength test for upper body strength, standing broad jump test for lower body strength and 20 m shuttle run test for cardiorespiratory endurance.

### Study procedures

Stool containers with unique identifiers were handed out to schoolchildren together with the instruction to return them with a small portion (at least 15 g) of their own morning stool. Containers were collected between 9 and 10 a.m. and transferred to a laboratory of the Nelson Mandela Metropolitan University (NMMU) in Port Elizabeth for diagnostic work-up on the same day. Stool samples were first visually examined for the presence of *Taenia* spp. proglottids, signs of blood, mucus and diarrhoea. Duplicate 41.7 mg Kato-Katz thick smears were prepared from each stool sample [21]. Slides were read under a microscope by experienced laboratory technicians who counted the number of eggs of each helminth species. The two slides were read by different technicians, the results compared for quality control and, in case of inconsistencies (i.e. positive *versus* negative or egg counts differing by more than 20 %), the slides were re-read. Helminth egg counts were multiplied by a factor of 24 to obtain a proxy for infection intensity, as expressed by the number of eggs per gram of stool (EPG) [22].

At the time of stool collection, children were given an empty urine collection container and asked to return it with a urine sample within the next 30 min. Filled containers were transferred to the laboratory and analysed on the same day. Samples were first inspected visually for macrohaematuria and then tested with Hemastix® strips (Siemens Healthcare Diagnostics GmbH; Eschborn, Germany) to detect blood in urine as a proxy for *Schistosoma haematobium* infections. A point-of-care circulating cathodic antigen (POC-CCA) urine cassette test (Rapid Medical Diagnostics; Cape Town, South Africa) was used for the diagnosis of *S. mansoni* infections [23].

For the detection of *C. parvum* and *G. intestinalis*, a Crypto-Giardia Duo-Strip® rapid diagnostic test (RDT) was performed on the stool sample, while for the discovery of *H. pylori*, a Pylori-Strip® RDT was employed (both tests from CORIS, BioConcept; Gembloux, Belgium).

The Hb concentration was measured once, to the nearest 0.1 g l^-1^, with the HemoCue® Hb 301 system (HemoCue®AB; Ängelholm, Sweden). In brief, after swabbing the child’s fingertip with alcohol, a field worker pricked the fingertip with a safety lancet and squeezed gently to obtain two drops of blood. The first drop was wiped away with the alcohol swab and the second drop was taken up with the microcuvette.

For the anthropometric measurements, each child was asked to remove the shoes and sweater before standing on a digital weighting scale (Micro T7E electronic platform scale, Optima Electronics; Georg, South Africa). Body weight was measured once to the nearest 0.1 kg. The height of each child was assessed with a Seca stadiometer (Surgical SA; Johannesburg, South Africa) whereby the child was standing with the back erect and shoulders relaxed. Body height was taken to the nearest 0.1 cm.

Specific standardised tests from the Eurofit fitness test battery [24] were conducted as follows. Upper body strength was determined through the grip strength test, with both right and left hands. Measurements were taken with the Saehan hydraulic hand dynamometer (MSD Europe BVBA; Tisselt, Belgium) set at handle position two. The examiner demonstrated how to grip the dynamometer with both arms at a 90° angle, while sitting straight and being relaxed. Each participant had three attempts, with about a 30 s rest in between, to grip the dynamometer with alternating hands as hard as possible. The maximum reading, measured to the nearest 1 kg, was recorded. The grip strength of both hands was measured. Additionally, the dominant hand was noted.

Lower body strength was estimated with the standing broad jump test. Before the start, the examiner demonstrated the test. Each child stood behind a straight line and jumped as far as possible with both legs forward. Participants had two attempts, with about a 30 s rest in between. The longer jump measured from the starting line to the heel of the foot closest to the starting line and rounded to the nearest 1 cm, was recorded.

The children’s endurance was measured with the 20 m shuttle run test [25], using the test protocol from Léger et al. [26] for which a great number of scientific international benchmarks exist [27, 28]. The 20 m flat grass running course was measured with a measuring tape and marked with different coloured cones. Five running lanes were created. The majority of the schoolchildren wore school or street shoes, whereas a minority ran barefoot. Shortly before the start of the test, the children were asked if anyone was sick or did not feel well. These children were excluded from the test. Next, the pre-recorded sound signals were played and the children did a trial run of two intervals (40 m). Once they were familiar with the test procedures, they were asked to run in groups, back and forth on the 20 m flat course, following the pace of the sound signals. Starting with a running speed of 8.5 km h^-1^, the frequency of the signal increased gradually such that every min, the pace increased by 0.5 km h^-1^. When a child failed to follow the pace in two consecutive intervals, she or he was asked to stop. The number of 20 m laps run to the last fully completed lap was noted as the final score.

### Statistical analysis

Data were double-entered, validated using EpiData version 3.1 (EpiData Association; Odense, Denmark) and merged into a single database. For children who had complete parasitological and anthropological data but missed one of the three physical fitness tests, we imputed data, using age- and sex-adjusted mean values. Statistical analysis was performed using STATA version 13.0 (STATA Corp.; College Station, TX, USA). Maps were created with ArcGIS version 10.2.1 (ESRI; Redlands, CA, USA).

Statistical significance was defined as *P* < 0.05. The parasitological status was described in terms of prevalence and infection intensity (mean EPG) of individual parasite species and the extent of multiparasitism (concurrent infections with more than one helminth or protozoan species). Anthropometric indicators, Hb concentrations and fitness performance scores were expressed as means and standard deviations (SD). Differences between groups were assessed using mixed linear models. The likelihood-ratio test was used to compare models. To describe the anthropometry of the children, body weight and height values were used to calculate the body mass index (BMI), defined as weight (in kg)/height^2^ (in m^2^), the sex-adjusted BMI-for-age Z-score (BMIZ) as an indicator for wasting and sex-adjusted height-for-age Z-score (HAZ) as an indicator for stunting [29].

The age of the participating child and the speed at which the child stopped running in the 20 m shuttle run test were converted into a third variable, the maximal oxygen uptake or VO_2_ max [25]. All statuses and indicators were compared between non-infected and infected children, the latter also further stratified by degrees of multiparasitism. Comparisons between schools were done using the χ^*2*^ test or the one-way ANOVA, as appropriate. Mixed linear and mixed logistic regression models with random intercepts for schools were used to analyse quantitative and binary data, respectively. These analyses included group comparisons with and without adjustment for covariates. For a simple interpolation of georeferenced data of children’s homes, the inverse distance weighting (IDW) method was used to obtain smoothed values of infection intensity, which is based on the assumption that two geographically close sites are more similar than two locations far apart.

## Results

### Demographic baseline characteristics

**Fig. 1:**
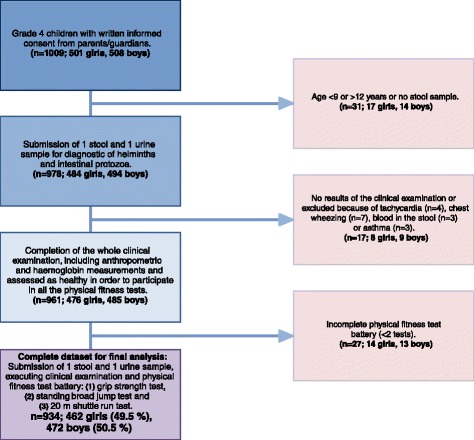
Study cohort and compliance of Grade 4 primary schoolchildren from eight schools in disadvantaged neighbourhoods of Port Elizabeth, South Africa in early 2015

All 1009 Grade 4 primary children of the eight selected schools from the northern part of Port Elizabeth were invited to participate. As illustrated in Fig. 1, complete data were available from 934 children (92 %). Reasons for exclusion were age outside the target range of 9–12 years, no stool or urine sample submitted for diagnostic work-up, lack of clinical examination, reported health problems precluding participation in the physical fitness tests (e.g. chronic asthma), or incomplete physical test battery. Children infected with either *C. parvum* or *G. intestinalis* and those who reported abdominal pain, blood in the stool or diarrhoea, those with special lung sounds (e.g. chest wheezing or creeping), ringworm infection or signs of tachycardia, were referred to the local clinic. All subsequent analyses refer to the final cohort of 934 children, which included 462 girls (49.5 %) and had a mean age of 10.0 years. No statistically significant difference was observed between the eight schools with regard to the mean age and sex ratio (both *P* > 0.05).

### Infections with helminths, intestinal protozoa and *H. pylori*

**Fig. 2:**
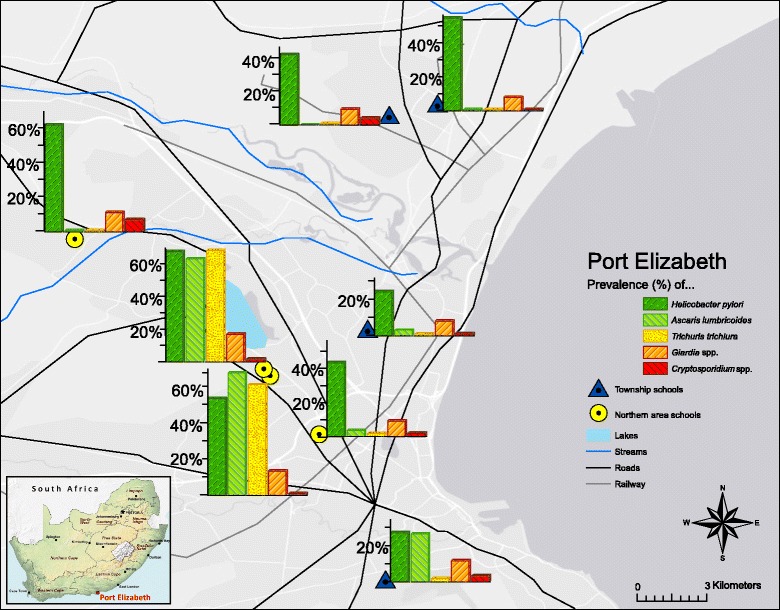
Prevalence of helminth, intestinal protozoan and *Helicobacter pylori* infection in eight primary schools in Port Elizabeth, South Africa, in early 2015

Overall, 248 children (26 %) were infected with *A. lumbricoides* and 207 (22 %) with *T. trichiura*. One child had a *S. haematobium* infection, while *Taenia* spp., hookworm and *S. mansoni* were not observed. A total of 144 children (15 %) were infected with at least one intestinal protozoan species: the *G. intestinalis* prevalence was 13 % and *C. parvum* prevalence was 3 %. *H. pylori* was found at all schools, ranging from 25 % up to 65 % (Fig. 2). Multiparasitism was common: 158 of the 384 infected children (41 %) harboured at least two parasite species, mostly *A. lumbricoides* and *T. trichiura*. Thirty triple-species infections were also detected (8 %).

**Fig. 3:**
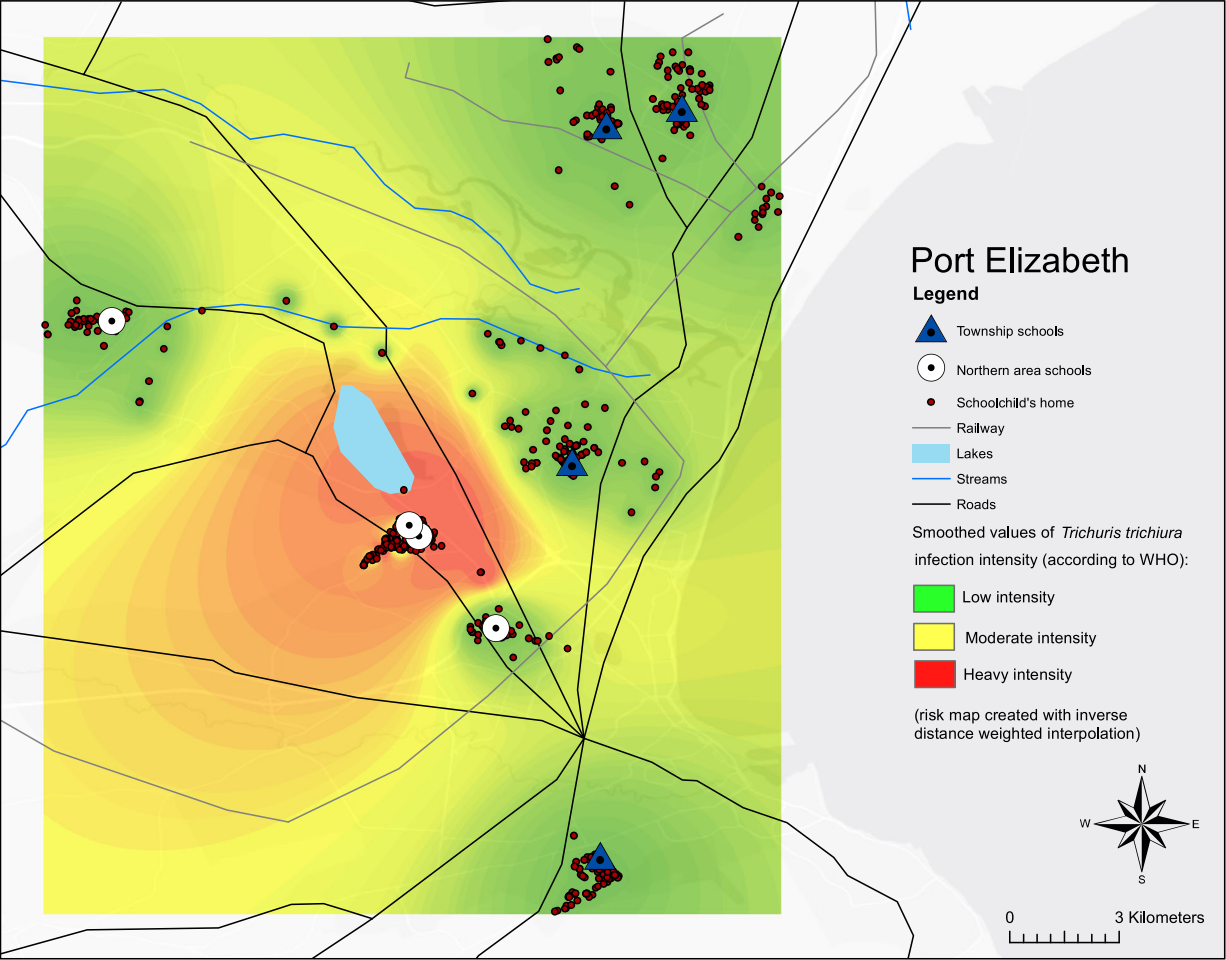
*Trichuris trichiura* infection intensities (stratified according to WHO guidelines) in the northern part of Port Elizabeth, South Africa, in February 2015, smoothed and based on 648 geographical coordinates of schoolchildren’s homes

Stratification by sex revealed that boys had higher prevalences and mean EPG values compared to girls for both *A. lumbricoides* and *T. trichiura* (Table 1). *T. trichiura* infections were of highest mean intensity in children aged 11 years (mean 940 EPG), while *A. lumbricoides* infections were of highest intensity in 12-year-old children (mean 18,630 EPG). Infections were spatially clustered: the prevalence of *T. trichiura* and *A. lumbricoides* at school B in Hillcrest was 65 and 60 %, respectively, and at school A in Helenvale 65 and 72 %, which was significantly higher than in the other schools (*T. trichiura*: *χ*^2^ = 592.53, *df* = 7, *P* < 0.0001 ; *A. lumbricoides*: *χ*^2^ = 475.34, *df* = 7, *P* < 0.0001) (Fig. 2). Similarly, infection intensities were highest in schools A and B (Fig. 3). At school B in Hillcrest and at school A in Helenvale, the prevalence of *G. intestinalis* was 16 and 14 %, respectively, and the respective prevalence of *H. pylori* was 65 and 57 %.

**Table 1:** *Ascaris lumbricoides* and *Trichuris trichiura* prevalence and infection intensity (as mean of duplicate Kato-Katz thick smears) among 934 primary schoolchildren from Port Elizabeth, South Africa, in early 2015, stratified by sex and age

		Sex			Age (years)				
		Male (*n* = 472)	Female (*n* = 462)		9 (*n* = 282)	10 (*n* = 375)	11 (*n* = 216)	12 (*n* = 61)	
		*n* (%)	*n* (%)	*P* ^a^	*n* (%)	*n* (%)	*n* (%)	*n* (%)	*P* ^a^
*A. lumbricoides*	Prevalence^b^	134 (28)	114 (25)	0.305	43 (15)	108 (29)	74 (34)	23 (38)	0.691
	Infection intensity^c ^								
	Mean EPG^d^ (95 % CI)	10,866 (7907–14,934)	9256 (6633–12,915)	0.333	8411 (4357–16,238)	9038 (6309–12,948)	10,899 (7306–16,258)	18,630 (11,725–29,602)	0.252
	Light (1–4999)	36 (8)	36 (8)		10 (4)	41 (11)	18 (8)	3 (5)	
	Moderate (5000–49,999)	67 (14)	58 (13)		26 (9)	43 (11)	39 (18)	17 (28)	
	Heavy (≥50,000)	31 (7)	20 (4)		7 (2)	24 (6)	17 (8)	3 (5)	
*T. trichiura*	Prevalence^e^	114 (24)	93 (20)	0.065	31 (11)	88 (23)	68 (32)	20 (33)	0.208
	Infection intensity^c^								
	Mean EPG^d^ (95 % CI)	757 (572–1002)	747 (557–1002)	0.950	737 (471–1155)	640 (467–877)	940 (661–1336)	744 (322–1723)	0.446
	Light (1–999)	65 (14)	55 (12)		16 (6)	55 (15)	36 (17)	13 (21)	
	Moderate (1000–9999)	44 (9)	35 (8)		15 (5)	32 (9)	27 (13)	5 (8)	
	Heavy (≥10,000)	5 (1)	3 (1)		0 (0)	1 (0.3)	5 (2)	2 (3)	

^a^All *P*-values are calculated using either mixed linear or mixed logistic regression, as appropriate, adjusted for clustering of schools

^b^ *A. lumbricoides* prevalence irrespective of co-infections

^c^ Stratified according to WHO guidelines

^d^ Geometric mean among the infected (95 % confidence interval)

^e^ *T. trichiura* prevalence irrespective of co-infections

### Anthropometric indicators and Hb concentration

The mean height, weight and BMI of the study cohort were 133.2 cm, 30.5 kg and 17.0 kg m^-2^, respectively. Stunting was observed in 10 % of the children, while wasting was recorded in 4 % of the children. We found statistically significant differences in anthropometric indicators when comparing children without a helminth infection with those with a single species infection of either *A. lumbricoides*, *T. trichiura* or a co-infection (Table 2). Non-infected children had greater body mass (*χ*^2^ = 11.09, *df* = 3, *P* = 0.011), height (*χ*^2^ = 11.60, *df* = 3, *P* = 0.009) and a higher BMI (*χ*^2^ = 9.49, *df* = 3, *P* = 0.024) and were less likely to be stunted (*χ*^2^ = 12.29, *df* = 3, *P* = 0.006) but not less wasted (*χ*^2^ = 2.83, *df* = 3, *P* = 0.418) compared to their peers with a single or dual species infection. Children concurrently infected with *A. lumbricoides* and *T. trichiura* had similar anthropometric and haemotologic measures compared to children with a single *T. trichiura* infection.

**Table 2:** Anthropometric indicators and haemoglobin concentrations among 934 primary schoolchildren, stratified by *Ascaris lumbricoides* and/or *Trichuris trichiura* infection status, from Port Elizabeth, South Africa, in February 2015

	Non-infected children	*A. lumbricoides* single infection	*T. trichiura* single infection	*A. lumbricoides-T. trichiura* co-infection	
	*n* = 635	*n* = 248	*n* = 207	*n* =156	*P* ^a^
Anthropometric					
Mean^b^ weight [kg] (95 % CI^c^)	31.6 (31.0–32.2)	28.4 (27.7–29.1)	27.8 (27.0–28.5)	27.8 (26.9–28.6)	0.011
Mean^b^ height [cm] (95 % CI^c^)	133.9 (133.3–134.5)	131.8 (130.9–132.6)	131.1 (130.1–132.1)	131.1 (130.0–132.2)	0.009
Mean^b^ BMI [kg m^-2^] (95 % CI^c^)	17.5 (17.2–17.7)	16.2 (16.0–16.5)	16.0 (15.7–16.3)	16.1 (15.7–16.4)	0.024
*n* (%) wasted^d^	15 (2.4)	17 (6.9)	16 (7.7)	12 (7.7)	0.418
*n* (%) stunted^e^	37 (5.8)	47 (19.0)	47 (22.7)	36 (23.1)	0.006
Haemotologic					
Mean^b^ haemoglobin [g l^-1^] (95 % CI^c^)	123.1 (122.3–123.8)	120.4 (119.2–121.6)	119.5 (118.3–120.6)	119.5 (118.2–120.8)	0.009

^a^All *P*-values are calculated using either mixed linear or mixed logistic regression, as appropriate, adjusted for clustering of schools

^b^Arithmetic mean

^c^95 % confidence interval

^d^Wasting is defined as ≤ -2 in BMIZ score

^e^Stunting is defined as ≤ -2 HAZ score

The overall mean Hb level was 122.2 g l^-1^. Hb levels were significantly lower in children harbouring a single or dual species helminth infection (*χ*^2^ = 11.70, *df* = 3, *P* = 0.009). In school B in Hillcrest, the mean Hb level was 119.7 g l^-1^, and at school A in Helenvale, 120.5 g l^-1^, respectively. These values were significantly lower compared to the overall mean Hb level of the eight schools enrolled in our study.

### Physical fitness levels and parasitological status

**Fig. 4:**
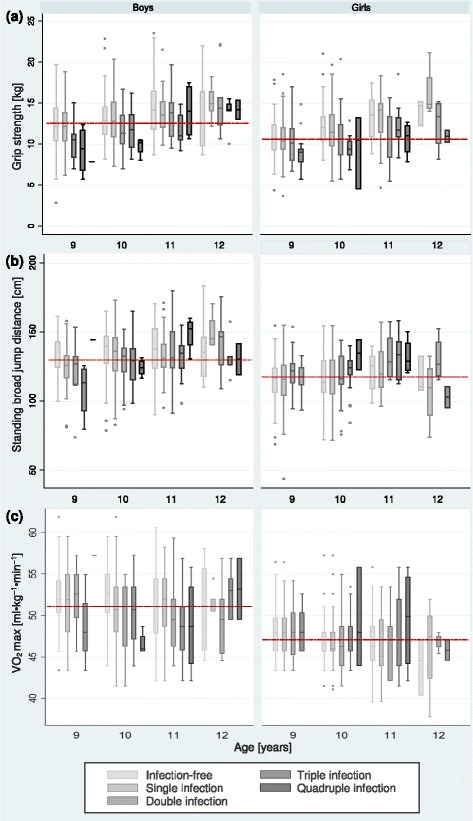
Physical fitness test results, namely (**a**) grip strength, (**b**) standing broad jump and (**c**) 20 m shuttle run test, among 934 Grade 4 schoolchildren, stratified by sex, age and infection status, in Port Elizabeth, South Africa, in early 2015. *Note*: The darker the boxplot is, the more parasite species are hosted by a child; *bright boxplots* represent infection-free, *black boxplots* represent quadruple infections; the *dashed red line* represents the mean

Non-infected boys achieved statistically significantly higher mean grip strength test results than non-infected girls (13.2 kg *versus* 11.7 kg; *χ*^2^ = 31.71, *df* = 1, *P* < 0.0001) (Fig. 4; Additional file 1: Table S1). Older children (11–12 years) had significantly higher mean grip strength test results compared to their younger counterparts (9–10 years) (*χ*^2^ = 150.25, *df* = 1, *P* < 0.0001). Irrespective of age and sex, children with multiple parasite infections had slightly, but not statistically significantly, lower mean grip strength compared to non-infected children.

Infection status was not associated with lower achievement in the standing broad jump test, irrespective of age and sex. The VO_2_ max estimated from the 20 m shuttle run test was higher in non-infected boys than girls (51.5 and 47.4 ml kg^-1^ min^-1^; *χ*^2^ = 167.43, *df* = 1, *P* < 0.0001), but unrelated to age.

With regard to infection with *A. lumbricoides* and *T. trichiura*, irrespective of the co-infection state (Table 3), the estimated mean VO_2_ max for 9-year-old children infected with *T. trichiura* was statistically significantly lower than the VO_2_ max of their non-infected peers (48.1 ml kg^-1^ min^-1^*versus* 49.6 ml kg^-1^ min^-1^; *χ*^2^ = 4.29, *df* = 1, *P* = 0.038). Estimates for infected children of higher age were lower compared to non-infected children of the same age, but the difference did not reach statistical significance.

**Table 3:** Mean maximal oxygen uptake (VO_2_ max) estimates^a^ (ml kg^-1^ min^-1^) among 934 primary schoolchildren from Port Elizabeth, South Africa, in February 2015, stratified by sex, age and *Ascaris lumbricoides* and *Trichuris trichiura* infection status

	*A. lumbricoides*			*T. trichiura*		
	Non-infected (*n* = 686)	Infected (*n* = 248)	*P* ^b^	Non-infected (*n* = 727)	Infected (*n* = 207)	*P* ^b^
Sex						
Male (*n* = 472) (95 % CI)	51.1 (50.7–51.5)	50.0 (49.3–50.6)	0.113	51.1 (50.7–51.5)	49.8 (49.1–50.6)	0.204
Female (*n* = 462) (95 % CI)	47.5 (47.2–47.8)	47.4 (46.7–48.0)	0.946	47.5 (47.1–47.8)	47.4 (46.7–48.1)	0.911
Age (years)						
9 (*n* = 282) (95 % CI)	49.6 (49.1–50.0)	48.6 (47.5–49.6)	0.104	49.6 (49.1–50.0)	48.1 (47.0–49.3)	0.038
10 (*n* = 375) (95 % CI)	48.9 (48.4–49.4)	48.8 (48.2–49.5)	0.955	48.9 (48.4–49.4)	48.9 (48.2–49.6)	0.778
11 (*n* = 216) (95 % CI)	49.2 (48.4–49.9)	48.8 (47.7–49.8)	0.489	49.3 (48.6–50.0)	48.4 (47.3–49.5)	0.151
12 (*n* = 61) (95 % CI)	49.9 (48.7–51.0)	49.0 (47.0–51.1)	0.424	49.2 (48.0–50.5)	50.2 (48.2–52.1)	0.548

^a^All mean VO_2_ estimates are expressed in ml kg^−1^ min^−1^ and are adjusted for age, with 95 % confidence intervals in parentheses when appropriate

^b^All *P*-values are calculated using either mixed linear or mixed logistic regression, as appropriate, adjusted for clustering of schools

In the multiple linear regression model presented in Table 4, sex and age were statistically significantly and negatively associated with mean VO_2_ max estimates. The mean VO_2_ max estimate of girls overall was 3.48 ml kg^-1^ min^-1^ lower than the VO_2_ max estimate of boys (*P* < 0.001). The mean VO_2_ max estimate also decreased by 0.40 ml kg^-1^ min^-1^ per year (*P* = 0.004). Non-significantly higher mean VO_2_ max estimates were found in *H. pylori*-infected children compared to their non-infected peers.

**Table 4:** Associations between mean maximal oxygen uptake (VO_2_ max) estimates (ml kg^-1^ min^-1^) and age, sex and infection status as predictor variables across eight schools. Data are derived from 934 primary schoolchildren from Port Elizabeth, South Africa, in early 2015

	Multiple linear regression		
Explanatory variables	Coefficient	95 % confidence interval	*P*
*A. lumbricoides* (reference: not infected)	-0.37	-1.23 to 0.50	0.403
Age (in years)	-0.40	-0.68 to -0.13	0.004
Dual infected (reference: not infected)	-0.42	-1.27 to 0.43	0.332
*T. trichiura* (reference: not infected)	-0.46	-1.61 to 0.70	0.442
Sex (reference: male)	-3.48	-3.95 to -3.01	< 0.001

*P*-value of mixed-effects linear regression model *P* < 0.001, adjusted for clustering within schools

## Discussion

We found notable levels of helminth and intestinal protozoa infections in 9- to 12-year-old children in eight schools of poor neighbourhoods in Port Elizabeth, South Africa. Children infected with *T. trichiura* had significantly lower body weight, were less tall and had a lower BMI compared to their non-infected peers (all *P* < 0.05). The same trend was observed for *A. lumbricoides*-infected children.

**Fig. 5:**
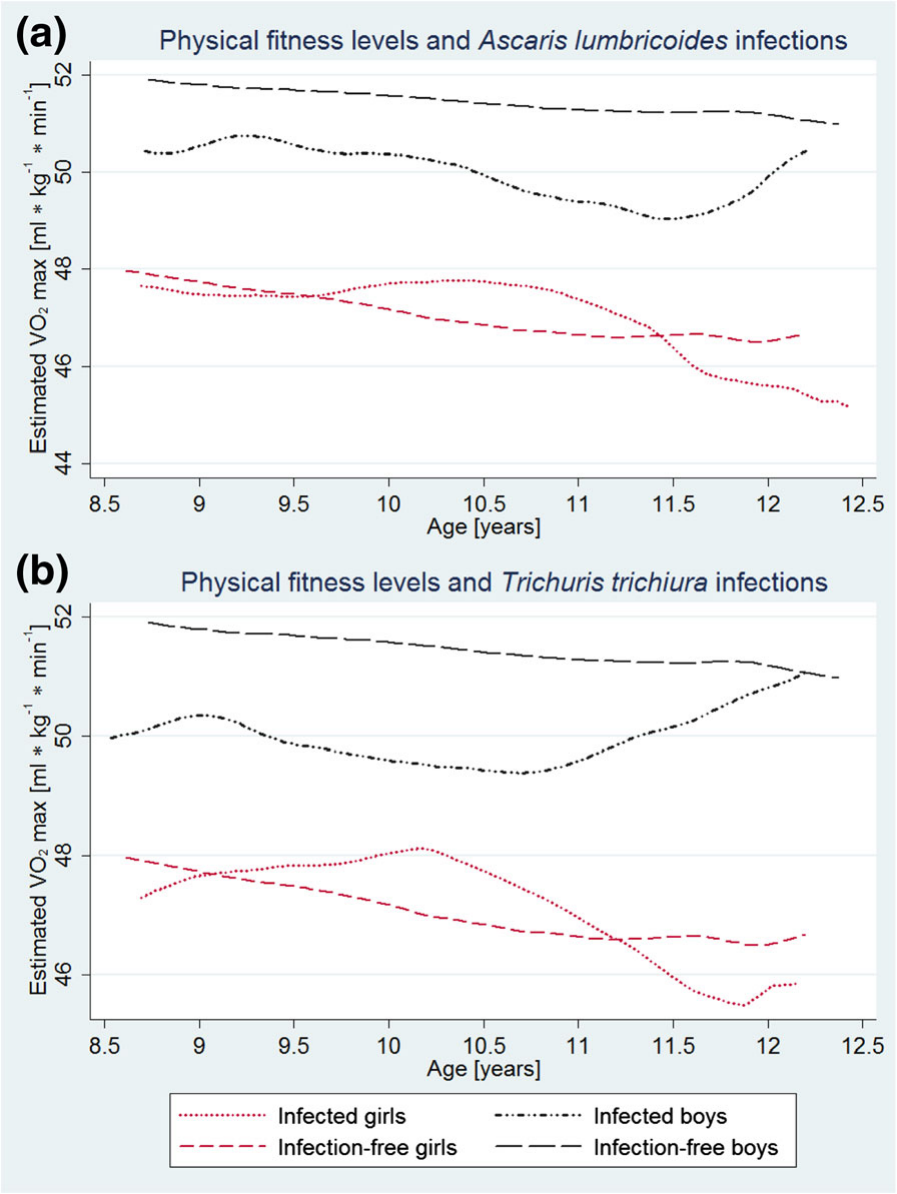
Physical fitness levels as estimated by maximal oxygen uptake (VO_2_ max) of children infected with either *A. lumbricoides* (*n* = 248) (**a**) or *T. trichiura* (*n* = 207) (**b**), compared to physical fitness levels of infection-free peers among Grade 4 schoolchildren in Port Elizabeth, South Africa, in early 2015. *Note*: Curves were generated using a polynomial smooth. Infection-free is here defined as no *A. lumbricoides*, *T. trichiura*, *Cryptosporidium* spp. and *Giardia* spp. infection (*n* = 278)

*Helicobacter pylori* infections were classified by World Health Organization (WHO) as a carcinogen of class 1 (definite carcinogen) in 1994 [30]. Non-significant associations between an infection with this bacterium and the growth of children were noted, confirming findings by Abdelrazak and Richter et al. [31, 32]. Boys, but not girls, with a *T. trichiura* or *A. lumbricoides* infection had significantly lower mean VO_2_ max estimates than non-infected peers (Fig. 5). Grip strength and standing broad jump test results were also statistically significantly lower in 9- to 10-year-old boys, whereas in girls no difference was seen.

The mean number of completed levels/stages of the 20 m shuttle run test corresponds closely to the mean results reported from other studies in different settings, e.g. the KISS- or the Sportcheck-study with Swiss primary schoolchildren of similar age [33–35]. Yap et al. reported slightly lower VO_2_ max results, 45.6 ml kg^-1^ min^-1^ for boys and 44.7 ml kg^-1^ min^-1^ for girls, from 194 children aged 9-12 years and living in south-west Yunnan province in the People’s Republic of China [3]. With regard to *T. trichiura* infections, Yap et al. [5] found more pronounced impacts on weight, height and BMI than the present study.

The 20 m shuttle run test was the selected measurement method for the assessment of cardio-respiratory fitness in a resource-constrained setting due to its ease of application [25]. An alternative test, though with smaller samples, is the Harvard step test (HST) [36, 37]. However, using different and technically more elaborated methods such as the cycle ergometer test used by Aandstad et al. [38], the estimated maximal oxygen uptake in 9- and 10-year-old children in Tanzania and Norway is significantly higher compared to the VO_2_ max of the 20 m shuttle run test (*P* < 0.001), namely for boys 58.6 ml kg^-1^ min^-1^ (95 % CI: 57.3–60.0 ml kg^-1^ min^-1^) and for girls 54.7 ml kg^-1^ min^-1^ (95 % CI: 52.9–56.5 ml kg^-1^ min^-1^) [38]. The estimated VO_2_ max values generated from the 20 m shuttle run test tend to be high in relation to other direct VO_2_ max measurement methods, such as maximal watt cycle ergometer test, treadmill or spirometry in laboratory settings. This shift in absolute level of VO_2_ max is not expected to influence the association signals with infection status, though.

Comparing calculated standing broad jump result means of 118 cm for girls and 132 cm for boys of the present survey with results of the Armstrong et al. study [39], also conducted in South Africa with 2819 girls and 3573 boys of the same age, Armstrong and colleagues measured noticeably longer standing broad jump distances, namely 152 cm for girls and 164 cm for boys.

The highest prevalence of stunting was observed in schools in the region of Hillcrest (22 %) and Helenvale (19 %), where also the highest prevalences of *A. lumbricoides* and *T. trichiura* were detected. However, these observations need to be interpreted with caution since current infection status is correlated with long-term growth indicators. Potentially, systematic differences in socioeconomic status and malnutrition levels exist between the study schools.

Only few studies have investigated the distribution of STHs in South Africa. Higher prevalences of hookworm and schistosome infections have been reported from warmer KwaZulu-Natal, located further north than Port Elizabeth [40, 41] compared to the results from our study. In our cohort of primary schoolchildren, heavy *T. trichiura* and *A. lumbricoides* intensities were observed in areas built in the 1950s to accommodate 6000 predominantly coloured people but where a recent survey estimated that more than 30,000 people are living in the area [39]. The area is characterised by unhygienic living conditions (poor sanitation and litter), high unemployment and gangsterism. Based on our results, biannual mass deworming should be implemented in the Hillcrest and Helenvale region in order to reduce STH prevalences and thus lower the risk of morbidity, complemented by interventions focusing on WASH [7].

Our study has several limitations. First, results reported here stemmed from a cross-sectional survey and as such we only identified associations rather than causality. Also, current infection status and current effects of past, long-term effects such as stunting are not directly linked. Secondly, it is still debated whether cardiorespiratory performance of children, measured here as maximal oxygen uptake (VO_2_ max), is receptive enough for change [42] due to varying personal living conditions. Thirdly, only single stool samples were collected from each participant. Hence, some infections, particularly those of light intensity, were possibly missed, as seen in other studies where multiple biological samples and a combination of diagnostic methods were employed [22, 43, 44]. Despite these limitations, the study confirms the practicability of the methods employed as suggested by previous experiences in different African and Asian settings, where school-aged children liked to perform physical fitness tests [45–47].

In the 2004 Global Burden of Disease (GBD) study, heavy *A. lumbricoides* and *T. trichiura* infections have both been assigned a zero disability weight (DW) as each of the two infections for itself alone is very rarely fatal, whereas the cognitive impairment resulting from both infections clearly differ, namely 0.463 for *A. lumbricoides* and 0.024 for *T. trichiura* on a scale from 0 (no disability) to 1 (death) [48]. In the GBD update 2013, a disease burden of 14.2 disability-adjusted life years (DALYs) per 100,000 person-years is estimated for children below the age of 15 years in South Africa who are infected with *A. lumbricoides*, while the respective estimate for children infected with *T. trichiura* is almost 10-fold higher (140 DALYs per 100,000 person-years) [49]. As we observed similar prevalences for *A. lumbricoides* and *T. trichiura* [41, 50], it appears that the disease burden of the latter helminth infection in under 15-year-old South African children is higher.

## Conclusions

This cross-sectional survey of the DASH study provides new insight into helminth and intestinal protozoa infections, physical fitness and growth of Grade 4 children in quintile three primary schools from disadvantaged communities in Port Elizabeth. Our results indicate that boys who are infected with multiple intestinal parasite species have lower physical fitness levels than their non-infected counterparts, as expressed by the maximal oxygen uptake (VO_2_ max). A significantly higher *T. trichiura* prevalence was noted in stunted children and those with a significantly lower Hb level, compared to children not infected with this species. Biannual mass deworming in order to control the morbidity due to STH infections is recommended in school B in Hillcrest and school A in Helenvale.

## Additional file

Additional file 1:
**Table S1.** Grip strength, standing broad jump and 20 m shuttle run test, among 934 Grade 4 schoolchildren from Port Elizabeth, South Africa, in early 2015, stratified by multiple infection status, sex and age. (DOCX 20 kb)

## Abbreviations

BMI: Body mass index
BMIZ: BMI-for-age Z-score
CI: Confidence interval
DALY: Disability-adjusted life year
DASH: Disease, Activity and Schoolchildren’s Health
DW: Disability weight
EKNZ: Ethics committee of Northwest and Central Switzerland
EPG: Eggs per gram (of stool)
GBD: Global Burden of Disease (study)
HAZ: Height-for-age Z-score
Hb: Haemoglobin
HST: Harvard step test
IDW: Inverse distance weighting
ISRCTN: International standard randomised controlled trial number
NMMU: Nelson Mandela Metropolitan University
NRF: National Research Foundation
POC-CCA: Point-of-care circulating cathodic antigen
RDT: Rapid diagnostic test
SD: Standard deviation
SNSF: Swiss National Science Foundation
SSAJRP: Swiss-South African joint research programme
STH: Soil-transmitted helminth
VO_2_ max: Maximal oxygen uptake
WASH: Water, sanitation and hygiene
WHO: World Health Organization
