# Supplementary material for: Intestinal parasites, growth and physical fitness of schoolchildren in poor neighbourhoods of Port Elizabeth, South Africa: a cross-sectional survey
Source: Parasit Vectors. 2016 Sep 5;9(1):488. doi: 10.1186/s13071-016-1761-5 (PMC5011914; doi:10.1186/s13071-016-1761-5)
Supplement: Additional file 1: — Table S1. Grip strength, standing broad jump and 20 m shuttle run test, among 934 Grade 4 schoolchildren from Port Elizabeth, South Africa, in early 2015, stratified by multiple infection status, sex and age. (DOCX 20 kb) [file 13071_2016_1761_MOESM1_ESM.docx]

## Additional file 1

## Table S1 Grip strength, standing broad jump and 20 m shuttle run test, among 934 Grade 4 schoolchildren from Port Elizabeth, South Africa, in early 2015, stratified by multiple infection status sex and age.

|  |  |  | **Sex** | |  | **Age (years)** | | |  |
| --- | --- | --- | --- | --- | --- | --- | --- | --- | --- |
|  |  |  | **Male**  **(n = 472)** | **Female**  **(n = 462)** |  | **9**  **(n = 282)** | **10**  **(n = 375)** | **11**  **(n = 216)** | **12**  **(n = 61)** |
|  |  |  | **Mean** ^a^ (n; SD ^b^) | **Mean** ^a^ (n; SD ^b^) |  | **Mean** ^a^ (n; SD ^b^) | **Mean** ^a^ (n; SD ^b^) | **Mean** ^a^ (n; SD ^b^) | **Mean** ^a^ (n; SD ^b^) |
| **(1) Grip strength test** | **Non-infected [kg]** | | **13.2** (135; 3.4) | **11.7** (143; 2.7) |  | **11.3** (96; 3.1) | **12.4** (107; 2.8) | **13.6** (57; 3.3) | **14.2** (18; 3.4) |
|  | **Infected [kg]** | |  |  |  |  |  |  |  |
|  | Single | | **12.9** (178; 2.9) | **11.7** (186; 3.0) |  | **11.3** (130; 2.6) | **12.3** (146; 2.8) | **13.2** (72; 3.0) | **16.0** (16; 3.2) |
|  | Double | | **12.7** (82; 3.2) | **10.9** (73; 2.9) |  | **10.5** (34: 2.6) | **11.3** (61; 2.9) | **12.9** (45; 2.9) | **14.2** (15; 3.8) |
|  | Triple | | **11.7** (69; 2.3) | **10.1** (51; 2.2) |  | **9.1** (21; 2.0) | **10.8** (54; 2.2) | **11.8** (36; 2.0) | **13.3** (9; 2.3) |
|  | Quadruple | | **12.6** (8; 3.5) | **10.1** (9; 2.7) |  | **7.8** (1; n.a.) | **9.8** (7; 2.6) | **12.8** (6; 3.7) | **12.9** (3; 2.4) |
| **(2) Standing broad jump test** | **Non-infected [cm]** | | **134.2** (135; 18.0) | **116.4** (143; 16.5) |  | **119.9** (96; 18.0) | **124.8** (107; 19.2) | **131.2** (57; 20.0) | **134.2** (18; 18.4) |
|  | **Infected [cm]** | |  |  |  |  |  |  |  |
|  | Single | | **130.4** (178; 17.3) | **116.2** (186; 17.6) |  | **119.5** (130; 18.5) | **123.7** (146; 18.8) | **126.1** (72; 17.6) | **134.5** (16; 22.9) |
|  | Double | | **131.3** (82; 17.9) | **122.4** (73; 16.3) |  | **122.7** (34; 15.9) | **122.7** (61; 17.6) | **133.1** (45; 16.5) | **137.5** (15; 17.3) |
|  | Triple | | **129.8** (69; 16.7) | **121.9** (51; 15.6) |  | **114.4** (21; 14.5) | **126.4** (54; 15.4) | **134.2** (36; 15.8) | **124.2** (9; 17.2) |
|  | Quadruple | | **132.1** (8; 12.6) | **128.0** (9; 16.0) |  | **144.5** (1; n.a.) | **128.6** (7; 9.3) | **134.9** (6; 12.9) | **118.3** (3; 23.5) |
| **(3) 20 m Shuttle run test** | **Non-infected [ml kg^-1^ min^-1^]** ^c^ | | **51.5** (135; 4.2) | **47.4** (143; 3.3) |  | **49.6** (96; 3.9) | **49.0** (107; 4.1) | **49.9** (57; 5.1) | **49.1** (18; 4.5) |
|  | **Infected [ml kg^-1^ min^-1^]** | |  |  |  |  |  |  |  |
|  | Single | | **50.9** (178; 4.0) | **47.5** (186; 3.1) |  | **49.6** (130; 3.6) | **48.8** (146; 4.2) | **48.9** (72; 4.0) | **49.8** (16; 3.7) |
|  | Double | | **50.1** (82; 3.7) | **47.2** (73; 3.3) |  | **49.1** (34; 3.7) | **48.7** (61; 3.7) | **48.3** (45; 4.2) | **48.9** (15; 3.6) |
|  | Triple | | **50.0** (69; 3.9) | **47.9** (51; 3.2) |  | **47.9** (21; 2.7) | **49.3** (54; 3.5) | **49.0** (36; 4.4) | **51.0** (9; 3.8) |
|  | Quadruple | | **49.1** (8; 5.4) | **48.3** (9; 5.5) |  | **57.2** (1; n.a.) | **48.0** (7; 3.8) | **47.7** (6; 5.8) | **49.5** (3; 7.4) |

^a^ **Arithmetic mean** (sample size (n); standard deviation (SD))
^b^All standard deviations (SD) are calculated using univariate analysis as appropriate
^c^ All mean VO_2_ estimates are expressed in ml kg^-1^ min^-1^  and are adjusted for age
n.a.: not applicable; n.r.: not represented
